# Effects of Perioperative Supplementation with Omega-3 Fatty Acids on Leukotriene B_4_ and Leukotriene B_5_ Production by Stimulated Neutrophils in Patients with Colorectal Cancer: A Randomized, Placebo-Controlled Intervention Trial

**DOI:** 10.3390/nu6104043

**Published:** 2014-09-29

**Authors:** Lone S. Sorensen, Ole Thorlacius-Ussing, Henrik H. Rasmussen, Søren Lundbye-Christensen, Philip. C. Calder, Karen Lindorff-Larsen, Erik B. Schmidt

**Affiliations:** 1 Department of Surgical Gastroenterology, Aalborg University Hospital, 9000 Aalborg, Denmark; E-Mail: otu@rn.dk; 2 Institute of Clinical Medicine, Aarhus University Hospital, Aarhus 8000, Denmark; 3 Center for Nutrition and Bowel Disease, Aalborg University Hospital, 9000 Aalborg, Denmark; E-Mail: hhr@rn.dk; 4 Department of Cardiology, Center for Cardiovascular Research, Aalborg University Hospital, 9000 Aalborg, Denmark; E-Mails: solc@rn.dk (S.L.); ebs@rn.dk (E.B.S.); 5 National Institute for Health Research Southampton Biomedical Research Center, University Hospital Southampton NHS Foundation Trust and University of Southampton, Southampton SO16 6YD, UK; E-Mail: p.c.calder@soton.ac.uk; 6 NordSim, Center for Simulation, Skills Training, Science and Innovation, Aalborg University Hospital, 9000 Aalborg, Denmark; E-Mail: kgll@rn.dk

**Keywords:** colorectal cancer, omega-3 fatty acids, immunomodulation, fish oil, leukotrienes

## Abstract

Omega-3 fatty acids (*n*-3 FA) may have beneficial clinical and immune-modulating effects in surgical patients. In a randomized, double-blind, prospective, placebo-controlled trial, 148 patients referred for elective colorectal cancer surgery received an *n*-3 FA-enriched oral nutritional supplement (ONS) providing 2.0 g of eicosapentaenoic acid (EPA) and 1.0 g of docosahexaenoic acid (DHA) per day or a standard ONS for seven days before surgery. On the day of operation, there was a significant increase in the production of leukotriene B_5_ (LTB_5_) (*p* < 0.01) and 5-hydroxyeicosapentaenoic acid (5-HEPE) (*p* < 0.01), a significant decrease in the production of leukotriene B_4_ (LTB_4_) (*p* < 0.01) and a trend for a decrease in the production of 5-hydroxyeicosatetraenoic acid (5-HETE) (*p* < 0.1) from stimulated neutrophils in the active group compared with controls. There was no association between LTB_4_ values and postoperative complications. In conclusion, oral *n*-3 FA exerts anti-inflammatory effects in surgical patients, without reducing the risk of postoperative complications.

## 1. Introduction

Patients undergoing surgery are at risk of developing complications in the postoperative period [1,2,3]. This is believed to be partly caused by changes in the immune response following surgery [4]. Thus, initially, a hyper-inflammatory response followed by a phase of relative immune incompetence occurs in relation to major surgery [5].

The pathophysiological changes are complex, but may be driven by excessive production of various lipid mediators, including the very potent pro-inflammatory leukotriene B_4_ (LTB_4_) produced from the omega-6 fatty acid (*n*-6 FA) arachidonic acid (AA) present in cell membranes. 

Among factors known to influence the clinical course of patients after surgery are nutritional status and specific biologically active nutrients [2,6,7,8,9,10] that might include the marine omega-3 fatty acids (*n*-3 FA) with the main biologically active *n*-3 FAs being eicosapentaenoic acid (EPA) and docosahexaenoic acid (DHA). Consumption of fish and fish oils oil increases the concentration of EPA and DHA in blood, cells and tissues [11,12] and alters the physical properties of cell membranes and the function of membrane proteins, including receptors, transporters and signalling proteins [13,14]. *n*-3 FA are incorporated into cell membranes in competition with the more abundant *n*-6 FA, AA, at the expense of the latter. AA may be liberated by phospholipases from cell membranes and induces leucocytes to produce the pro-inflammatory LTB_4_ and the side product, 5-hydroxyeicosatetraenoic acid (5-HETE). In contrast, leukotriene B_5_ (LTB_5_) and the side product, 5-hydroxyeicosapentaenoic acid (5-HEPE), derived from EPA [15], have considerably less potent biological activities in comparison to LTB_4_ [15,16]. Replacement of *n*-6 FA with *n*-3 FA in membranes of immune active cells may therefore lead to reduced formation of pro-inflammatory compounds, and by this, and other [5,12] mechanisms, *n*-3 FA may decrease infectious complications after surgery [7,17,18,19,20]. The influence of enteral feeds, including *n*-3 FA on AA-derived eicosanoids (e.g., LTB_4_), has been the subject of much attention [5,21,22,23]. Several studies have indicated that *n*-3 FAs modulate the generation of inflammatory eicosanoids in gastrointestinal surgical patients [24,25,26] and may help to counteract the surgery-induced decline in antigen-presenting cell activity [20] and T-cell cytokine production [27].

The aim of the present study was to evaluate the production of LTB_4_, 5-HETE, LTB_5_ and 5-HEPE from stimulated neutrophils after seven days of preoperative treatment with an *n*-3 FA-enriched oral nutritional supplement (ONS) in patients undergoing colorectal cancer surgery and to study the possible impact on clinical outcome. Furthermore, the correlation between LTB_4_ values and postoperative complications was investigated.

## 2. Materials and Methods

### 2.1. Study Design

**Figure 1:**
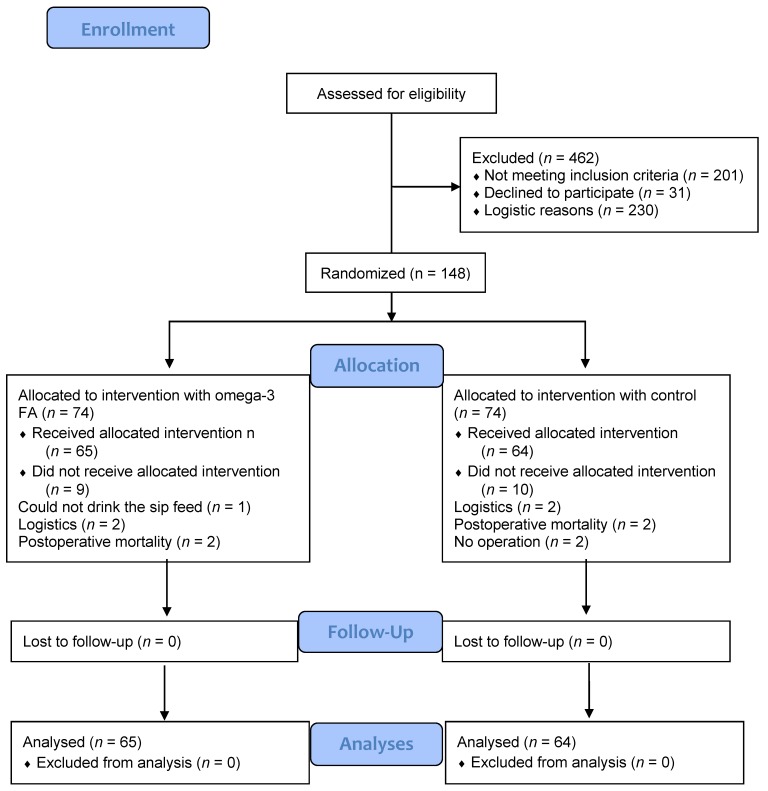
Patient flow through the study.

This was a sub-study of a randomized, double-blind, prospective, placebo-controlled single-centre interventional trial involving 148 participants (Figure 1) awaiting colorectal cancer surgery [28]. Participants were recruited consecutively from the outpatient clinic of the Department of Surgical Gastroenterology, Aalborg University Hospital. All eligible participants were asked to participate.

Exclusion criteria were diabetes mellitus, consumption of >5 alcoholic drinks per day, emergency surgery, inability to understand the spoken and written information in Danish, untreated psychiatric conditions, pregnancy or breast-feeding, reduced kidney function (plasma creatinine > 130 μmol/L), use of *n*-3 FA supplements, anticipated poor compliance, immunosuppressive diseases and participation in another clinical trial.

After providing oral and written informed consent, participants were randomly assigned to treatment with *n*-3 FA (active treatment) or control (a standard ONS without marine *n*-3 FA), 200 mL twice per day (morning and afternoon) for 7 days before surgery. Randomization was performed using sealed non-transparent envelopes containing the randomization number and kept at the investigation site according to CONSORT (Consolidated Standards of Reporting Trials) guidelines [29]. The active and the control ONS cartons looked identical and had identical taste and scent (coffee). The main investigator and a study nurse enrolled the participants at the outpatient clinic and randomly assigned them to active treatment or control. The participants, carers, investigators and other researchers were blinded to treatment allocation throughout the study. The investigators had no access to the code until after completion of the study. Statistical analyses were completed before the code was broken.

Information collected included demographic data, tumour location and American Society of Anaesthesiologists (ASA) risk score [30]. All patients underwent standard Nutritional Risk Screening (NRS 2002) [31]. A food questionnaire focusing on consumption of seafood on a monthly basis was completed at baseline. 

The study was approved by the regional ethics committee (N-VN-20050035) and conducted according to the Hong Kong amendment to the Declaration of Helsinki. The trial was registered at ClinicalTrials.gov: ID NCT00488904 [32].

### 2.2. Intervention

Participants in the active or control group received the ONS as a sip feed (200 mL twice a day morning and afternoon) for 7 days before surgery. The feeds (Supportan^®^) were isocaloric (1.5 kcal/mL) and isonitrogenous (Table 1) and were provided by Fresenius Kabi (Bad Homburg, Germany). Both feeds contained the same amounts of carbohydrate, protein, total fat and *n-*6 FA (Table 1).

**Table 1:** Daily intake of energy and nutrients from the *n*-3 FA-enriched and the control oral nutritional supplement.

Daily dose	Control	*n*-3 FA (Active)
Energy (kcal)	600	600
Protein (g)	40	40
Carbohydrate (g)	49.6	49.6
Fat (g)	26.8	26.8
EPA (g)	0	2
DHA (g)	0	1
Total *n*-6 FA (g)	3.3	3.3

Fat content of the supplement was comprised of medium chain triglycerides, sunflower oil and safflower oil. The active supplement also contained additional fish oil at a level to achieve 2 g EPA and 1 g DHA per day (Table 1). Participants were provided with the sip feeds at inclusion, to consume twice a day for 7 days at home before hospitalization. A questionnaire regarding compliance preoperatively was completed, and good compliance was defined as self-reported consumption of at least 12 of the 14 ONS cartons before surgery.

### 2.3. Isolation of Blood Neutrophils

Blood was drawn in the fasting state on the day of the surgery. Neutrophils were separated from anticoagulated (K-EDTA 1.6 mg/mL) blood layered on top of PolymorphprepTM (AXIS-SHIELD PoC AS, Rodeloekka, Norway) and separated by a one-step centrifugation technique at 450 g for 40 min. Neutrophils were harvested and washed twice in tissue culture medium (RPMI 1640, Sigma-Aldrich, Ayrshire, UK), at ambient temperature and centrifuged for 10 min at 520× *g*. Subsequently, neutrophils were counted and red cells eliminated by the addition of ice-cold 0.2% saline for 35 s. Next, 1.6% ice-cold saline was added in order to obtain an isotonic 0.9% concentration, followed by centrifugation at 300× *g* at 5 °C for 5 min, which was repeated once. Neutrophils were then washed in a phosphate buffer containing glucose and human albumin (PBS) and resuspended in PBS adjusting the concentration to 1 × 10^7^ neutrophils/mL PBS. Isolated neutrophils were stored at −80 °C until analysis.

### 2.4. Analysis of Leukotrienes, 5-HEPE and 5-HETE

The neutrophil suspension (0.9 mL of 1 × 10^7^ granulocytes/mL PBS) was prewarmed to 37 °C, and CaCl_2_, MgCl_2_ and calcium ionophore (A23187) at a final concentration of 10 µM were added to initiate stimulation. After 10 min, the reaction was terminated by the addition of 100% ice-cold ethanol, and the mixtures were centrifuged at 4 °C at 700× *g*. The supernatant was stored at −80 °C for later analysis. C18 cartridges (Sep-Pak VAC RC, Waters Co., Milford, MA, USA) were used for the extraction of leukotrienes (LT), 5-HEPE and 5-HETE. The ethanol mixture was thawed and centrifuged, and international standard prostaglandin B_2_ (PGB_2_) and trifluoroacetic acid were added. The cartridges were conditioned and equilibrated using methyl formate, 100% ethanol and water. The acidified sample was loaded onto the cartridge, washed with 15% ethanol, water and hexane and eluted with methyl formate. The solvent was evaporated to dryness under nitrogen, and the sediment was dissolved in the mobile phase (31% H_2_O, 27% methanol, 42% acetonitrile and 0.025% trifluoroacetic acid).

Analysis was performed by high pressure liquid chromatography (Dionex Ultimate LPG-3400A) on an Acclaim RSLC 2.1 mm × 100 mm C18 column (Dionex Corporation, Sunnyvale, CA, USA). Concentrations were calculated using the internal standard and response factors. The response factors were calculated by analysis of a non-stimulated neutrophil suspension after the addition of known amounts of standards of LTB_4_, LTB_5_, 5-HETE and 5-HEPE, as well as an internal standard (PGB_2_). Samples were extracted and analysed using high performance liquid chromatography, and eventually, the recovery factors were calculated.

### 2.5. Granulocyte Fatty Acid Analysis

Blood was drawn in the fasting state on the day of surgery. Granulocytes were prepared as described previously [33]. FA profiles were determined by gas chromatography using a Varian 3900 gas chromatograph, CP-8400 autosampler and CP 8414 autoinjector (Varian, Middelburg, The Netherlands), as well as a flame ionization detector. In split injection mode, a CP-sil 88.60-m × 0.25-mm capillary column (Varian, Middelburg, The Netherlands), temperature programming from 90 to 205 °C, a constant flow rate of 1.0 mL/min and helium carrier gas were used. Results for individual FAs are expressed as a percentage of the total FA content.

### 2.6. Statistical Analysis

The basic characteristics of the trial population were analysed with Fisher’s exact test for categorical variables and unpaired *t*-tests for continuous variables. Differences between treatment groups were analysed using unpaired *t*-tests. If variances differed between groups, Welch’s approximation was used. The distribution of continuous data was analysed for normality. As the values for 5-HEPE, 5-HETE and LTs were right skewed distributed, log transformed observations were analysed, and these were normally distributed. Distributions of 5-HEPE, 5-HETE and LT were described with median and inter-quartile range (IQR). Relative differences between groups in medians were calculated by exponentiation of the differences between log-transformed means. Associations between LTB_4_ values and postoperative complications were analysed using logistic regression. The associations between log-transformed LT values 5-HEPE and AA, EPA and AA/EPA were analysed using linear regression. Analyses were performed blinded to treatment groups. The active and control groups were not identified until after the statistical analyses had been conducted. All *p*-values were two-tailed, and differences were reported with 95% confidence intervals (CI). *p*-values below 0.05 were considered significant. All analyses were performed using Stata version 11.2 (StataCorp, 2009; Texas City, TX, USA).

## 3. Results

### 3.1. Participants Characteristics

All eligible participants (*n* = 610) were asked to participate, but 230 participants were not included. This was due to a change in clinical practice during the study, such that many patients were offered surgery within a five-day period, which did not allow for participants to complete the seven-day intervention. Furthermore, some participants did not meet the inclusion criteria (201), and 31 participants declined to participate. Baseline characteristics of the included *vs.* the non-included participants did not differ.

A total of 148 consecutive patients (68 females, 80 males; mean age 71 (range 41–89) years) were included in the study. The majority of participants had open surgery; laparoscopic resection was only performed in nine patients in the control group and nine in the *n*-3 FA group. Participant characteristics did not differ between treatment groups (Table 2).

**Table 2:** Characteristics of patients in the control and active groups.

Variable	Control (*n* = 74)	Active (*n* = 74)	*p*
**Demographic data**			
Age, years, mean (SD)	71 (10)	69 (11)	0.164
Sex (male/female)	36/38	44/30	0.248
Body weight, kg, mean (SD)	76 (19)	77(17)	0.570
Height, cm, mean (SD)	169 (11)	171 (9)	0.301
BMI, kg/m^2^, mean (SD)	26 (5)	26 (5)	0.651
Weight loss * (*n*)	19	11	0.068
**Clinical characteristics**			
Smoking/non-smoking (*n*)	11/60	17/54	0.292
Unknown smoking status	3	3	
**Cancer location**			
Colon/rectum (*n*)	40/34	38/36	0.869
**Surgical procedure**			0.977
Right hemicolectomy + transverse colon (*n*)	16	17	
Left hemicolectomy + sigmoid colon (n)	10	12	
Laparoscopic resection of sigmoid colon (*n*)	9	9	
Low anterior resection of rectum or abdominoperineal resection	28	30	
Colectomy (*n*)	4	3	
Other rectum resection (n)	7	3	
Nutritional status **			0.089
No risk (NRS score <3) (*n*)	23	34	
At risk (NRS score ≥3) (*n*)	50	39	
Unknown (*n*)	1	1	

Notes: There were no significant differences between groups (*p* > 0.068); BMI, body mass index; * defined as loss of more than 5% of body weight; ** defined according to NRS 2002 [31].

### 3.2. Fatty Acid Composition of Neutrophils

Neutrophil EPA and DHA were significantly higher, and AA and linoleic acid was significantly lower in the group receiving *n*-3 FA than in the control group (Table 3) [28]. 

The food questionnaire indicated an average dietary intake of *n*-3 FA of 0.6 g per day, with no difference in preoperative intake between groups (*p* = 0.770). None of the included participants received more than 150 mg of anti-inflammatory drugs daily. Both supplements were well tolerated with no adverse effects reported. Nine participants randomized to active treatment and 10 participants in the control group did not receive the allocated intervention for reasons listed in Figure 1.

Preoperatively, 63 of 65 participants in the active group were compliant compared with 56 of 64 participants in the control group (*p* = 0.266). Two participants died in each group. In the active group, death was caused by pneumonia and a myocardial infarction, whereas the participants in the control group died from septicaemia and sudden cardiac death.

**Table 3:** Granulocyte fatty acids on day of operation in the control and active groups.

	Weight% of Total FA Content	
	Control	Active
EPA	0.54 (0.42–0.74)	2.10 (1.83–2.55) *
DPA	0.54 (0.42–0.74)	2.11 (1.83–2.55) *
DHA	1.31 (1.10–1.57)	1.61 (1.34–1.84) *
Total *n*-3 FA	2.44 (1.98–2.97)	5.95 (5.20–6.75) *
Arachidonic acid	12.51 (11.65–13.19)	11.61 (10.67–12.48) *
Linoleic acid	8.94 (8.24–9.49)	9.42 (8.72–10.19) **

Notes: Values are the median (IQR); FA, fatty acid; ONS, oral nutritional supplement; EPA, eicosapentaenoic acid; DPA, docosapentaenoic acid; DHA, docosahexaenoic acid; * *p* < 0.001; ** *p* < 0.05 *versus* the control group.

### 3.3. Production of Mediators from Neutrophils

Furthermore, compared to neutrophils from controls, those from participants in the *n*-3 FA group showed a significantly higher (by 176% and 306%, respectively) production of LTB_5_ (*p* < 0.001) and 5-HEPE (*p* < 0.001) (Table 4).

**Table 4:** Formation of leukotrienes (LT) and side products (5-HEPE; 5-HETE) from activated neutrophils according to treatment group.

Eicosanoids	Control	Active	% Difference
LTB_5_	5.8 (4.9–7.6)	17.5 (13.5–22.8)	176 *** (143–215)
LTB_4_	186.8 (156.8–230.7)	163.5 (136.6–199.9)	−12 *** (−21–−3)
5-HETE	293.1 (246.5–357.8)	273.7 (221.8–320.7)	−7 * (−14–−0)
5-HEPE	34.2 (25.7–55.3)	154.7 (122.4–190.4)	306 *** (255–364)
LTB_4_/LTB_5_	31.8 (25.0–40.1)	9.6 (7.8–11.4)	−68*** (−72–−65)

Notes: Data are the median (IQR) ng/10^7^ neutrophils and the percentage of difference between estimated medians; *** indicates *p* < 0.01; * indicates *p* < 0.1; ng/10^7^ = nanogram/10^7^ neutrophils.

Conversely, in the active group, neutrophils showed a significantly lower (by 12%) production of LTB_4_ (*p* < 0.001) and a trend towards lower (by 7%) production of 5-HETE (*p* = 0.059). LTB_4_/LTB_5_ was significantly different between groups (by 68%) (*p* < 0.001) (Table 4). There was no statistically significant difference in clinical outcomes (total number of complications, infectious complications, non-infectious complications, intensive care unit stay, mortality, readmissions and hospital stay) between groups, as reported previously [28].

There was no statistically significant association between the values of the proinflammatory LTB_4_ production and any clinical outcome, including total number of complications (*p* = 0.524), infectious complications (*p* = 0.660) and non-infectious complications (*p* = 0.307) (Table 5). The ratio LTB_4_/LTB_5_ did not have a statistically significant association with the total number of complications (*p* = 0.707), infectious complications (*p* = 0.711) and non-infectious complications (*p* = 0.143) (Table 4).

**Figure 2:**
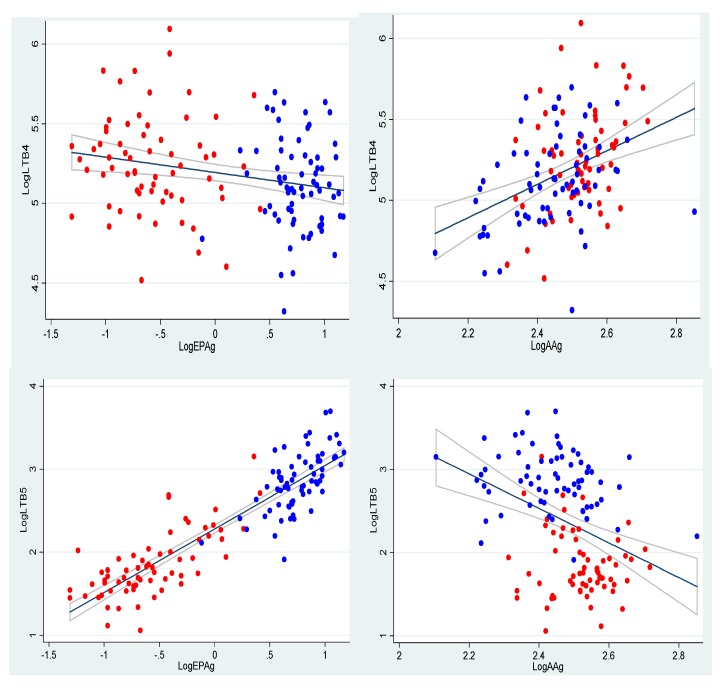
Associations between log-transformed AAg (AA content in the cell membranes of the granulocytes) and EPAg (EPA content in the cell membranes of the granulocytes) in the neutrophils, as well as the formation of LTs (LTB_4_ and LTB_5_ (ng/10^7^)) by neutrophils, illustrated using scatter plots with regression lines and confidence bands added. Control group, red dots; active group, blue dots.

However, There were strong associations between the content of AA and EPA in neutrophils and production of LTB_4_ and LTB_5_ (Figure 2) (all *p* < 0.01).

These graphs illustrate that the higher the content of EPA in the cell membranes, the higher the production of LTB_5_. Furthermore, it can be seen that the higher the content of AA in cell membranes, the lower the production of LTB_5_. Furthermore, there were strong associations between AA/EPA in neutrophils and LTB_4_ and LTB_5_ production (both *p* < 0.01) (results not shown) and between AA, EPA, AA/EPA and 5-HEPE production (all *p* < 0.001) (results not shown).

**Table 5:** Associations between LTs production by neutrophils and clinical outcome described by odds ratio (OR), CI and *p*-values for a unit change in LTB_4_ and LTB_4_/LTB_5_, respectively.

	LTB_4_			LTB_4_/LTB_5_		
	OR	CI	*p*	OR	CI	*p*
Infectious complications	1.00	1.00–1.01	0.660	1.00	0.97–1.03	0.711
Non-infectious complications	1.00	0.99–1.00	0.307	0.98	0.94–1.00	0.143
Total number of complications	1.00	0.99–1.00	0.524	1.00	0.98–1.03	0.707

## 4. Discussion

In this prospective randomized, double-blind, single-centre, placebo controlled study, it was demonstrated that seven days of enteral supplementation with 3 g of EPA + DHA daily resulted in a higher neutrophil production of LTB_5_ and a lower production of LTB_4_ compared to the control group. In addition, there was a higher neutrophil 5-HEPE production and a trend to lower production of 5-HETE in the active group compared to the control. While the differences in LTB_4_ and LTB_5_ production indicate an anti-inflammatory action of the supplement, it is unknown whether the difference in formation of 5-HETE and 5-HEPE between groups is of clinical relevance. However, 5-HETE enhances lymphocyte proliferation, whereas 5-HEPE only has one-tenth the potency of 5-HETE regarding this [34]. Thus, the current study demonstrates that preoperative supplementation with *n*-3 FA for one week can modulate immune function, assessed as the production of lipoxygenase mediators, in participants admitted for elective colorectal cancer surgery. However, this was not associated with a decrease in postoperative complication rates [28].

The strengths of the present study are that it was a randomized, prospective, relatively large clinical study, was double-blind, with an identical appearance for the sip feed cartons, the taste of *n*-3 FA was undetectable and that compliance was acceptable. Furthermore, the study population was relatively homogenous.

One limitation of the present study is that we only had data on eicosanoid formation from the day of surgery, whereas postoperative changes would also have been of interest. The short duration of the intervention (seven days) may have limited the incorporation of *n*-3 FA and the decrease of AA in neutrophils and, thereby, limited the impact on LTB_4_ production, but a longer period of supplementation was not possible, as participants were operated on soon after cancer diagnosis. However, we showed in earlier publications that 3 g of *n*-3 FA for seven days before surgery was sufficient to assure significant incorporation of *n*-3 FA into neutrophils and into colonic tissue [28,35]. The required sample size was based on a reduction in postoperative infection rates from 30% to 10% and was calculated to be 148 participants in all, but we were only able to analyse data from 129 of these. A final limitation is the discrepancy between the number of eligible (610) and analysed participants (129) due to a change in clinical practice during the study, such that many patients were offered surgery within a five-day period, which did not allow for participants to complete the seven-day intervention.

Our findings are consistent with the results from three recent studies in humans [24,36,37]. In a prospective double-blind study, Wang *et al.* [37] randomized 64 participants with a need for postoperative parenteral nutrition after surgery into two groups. The study population was a mix of surgical patients (22 gastric cancers; 29 colonic cancers; 13 with other digestive diseases). They received either fish oil containing lipid emulsion (a mixture of soybean oil, MCT and fish oil) as part of the intravenous regimen, or a mix of soybean oil and MCT for 5 days after surgery. There was a significant increase in the neutrophil LTB_5_/LTB_4_ ratio but no effect on clinical outcome, infectious complications and bleeding events. Grimm *et al.* [36] randomized 33 participants undergoing major abdominal surgery into two groups in a prospective double-blind study to receive parenteral nutrition providing either a fish oil containing lipid emulsion (a mixture of soybean oil, MCT, olive oil and fish oil) or soybean oil for five days after surgery. The study population was again a mix of surgical patients. The initial production of LTB_4_ and LTB_5_ by neutrophils was similar in both groups. The production of LTB_5_ from neutrophils was significantly increased, and the release of LTB_4_ was decreased, though not significantly, in the participants receiving fish oil. The length of hospital stay was significantly shorter in the intervention group. Finally, Köller *et al.* [24] conducted a prospective double-blind randomized study with 30 participants undergoing colorectal surgery. Participants received parenteral nutrition, providing either a fish oil containing lipid emulsion (a mixture of soybean oil, MCT and fish oil) or soybean oil for five days post-surgery. This study also found a significant increase in LTB_5_ production by leukocytes in the fish oil group, but without a concomitant decrease in LTB_4_ production. These three studies all made use of intravenous (IV) nutrition given postoperatively. The present study, which used enteral nutrition given preoperatively, agrees with these earlier findings of increased LTB_5_ production and decreased LTB_4_ production after fish oil provision, but with limited clinical impact.

Some earlier studies have reported beneficial effects of oral *n*-3 FA supplementation in gastrointestinal surgery patients. Wachtler *et al.* [26] analysed leukocyte function in 40 participants undergoing major upper gastrointestinal surgery in a placebo-controlled double-blind study. One group received an *n*-3 FA-enriched (0.33 g/100 mL) oral supplement, also containing arginine and ribonucleic acid, and the other group received a standard control supplement for five days preoperatively. There was a significantly higher production of LTB_5_ from neutrophils in the intervention group when compared to controls. However, no changes in LTB_4_ were evident in the intervention group. The authors reported a low number of postoperative complications. In another study, Shimizu *et al.* [38] gave 12 children with ulcerative colitis 1.8 g EPA orally per day for two months. LTB_4_ production by leucocytes and colonic mucosa were assessed before and after the intervention. Biopsies were taken from the rectal mucosa during sigmoidoscopy before and after initiation of EPA supplementation. After two months of supplementation, there was a decrease in LTB_4_ production by leucocytes and colonic mucosa, while no information regarding LTB_5_ production was given.

IV administration of *n*-3 FA ensures a quicker incorporation of the presumed active substances (*n*-3 FA) into the membranes of immune cells [39,40]. However, this format is not feasible in the pre-operative setting. ONSs are less expensive and enable the use of the gut prior to surgery. Two studies providing IV *n*-3 FA for five days post-operatively did not show any decrease in the production of LTB_4_ [24,36]. Importantly many of these studies did not provide explicit information about the amount of *n*-3 FA given. 

In the present study, seven days of oral supplementation with 3 g of *n*-3 FA daily ensured significant incorporation of EPA into neutrophils and a significant decrease in the formation of LTB_4_. However, to achieve an anti-inflammatory response meditated by *n*-3 FA, it is probably more important that the formation of the AA-derived LTB_4_ is suppressed than an increase in LTB_5_. Despite this, there was no effect on clinical outcome in the current study. One explanation for this may be that *n*-3 FA incorporation was not sufficiently high. A higher *n*-3 FA dose or a longer duration of intervention could have had an impact on clinical outcome. The ratio LTB_4_/LTB_5_ was 68% lower in the active group, which is a considerable decrease, but still did not have any effect on clinical outcome. 

One other important factor may be that the nutritional status of the patients, evaluated by NRS 2002, was generally good for most participants. A weight loss of more than 5% of body weight was only detected in 23% of participants entering the study. This could account for the lack of clinical improvement with the *n*-3 FA-enriched ONS, since it is likely that malnourished participants might benefit the most from ONS and from oral *n*-3 FA.

## 5. Conclusions

In summary, the current study shows that an ONS providing 3 g of *n*-3 FA daily for seven days before surgery was able to induce a significant decrease in the formation of the pro-inflammatory LTB_4_ from neutrophils with a simultaneous increased production of LTB_5_. A decrease in the formation of 5-HETE, though not significant, and a significant rise in 5-HEPE was also seen. However, the clinical consequences of these changes are unknown. Associations between values of LTB_4_ or LTB_4_/LTB_5_ and postoperative complication rates were not seen. This indicates either that the changes observed were too small or that the formation of LTs from activated neutrophils is not an important determinant of surgical complications. Whether a longer period (months) of *n*-3 FA intake could be of a benefit for patients operated on for colorectal cancer regarding shorter stay in hospital or longer survival needs to be investigated in larger trials.

## Abbreviations

AA: arachidonic acid
ASA: American Society of Anaesthesiologists
BMI: body mass index
CI: confidence intervals
DHA: docosahexaenoic acid
EPA: eicosapentaenoic acid
5-HEPE: 5-hydroxyeicosapentaenoic acid
5-HETE: 5-hydroxyeicosatetraenoic acid
IV: intravenous;
IQR: inter-quartile range
LT: leukotrienes
LTB_4_: leukotriene B_4_
LTB_5_: leukotriene B_5_
*n*-3 FA: *n*-3 fatty acids
*n*-6 FA: *n*-6 fatty acids
MCT: medium-chain triglycerides
ng: nanograms
NRS 2002: Nutritional Risk Screening
ONS: oral nutritional supplement
OR: odds ratio
PBS: phosphate-buffered saline
PGB_2_: prostaglandin B_2_
